# A multiparametric method to assess the MIM deformable image registration algorithm

**DOI:** 10.1002/acm2.12564

**Published:** 2019-03-28

**Authors:** Silvia Calusi, Giusy Labanca, Margherita Zani, Marta Casati, Livia Marrazzo, Linhsia Noferini, Cinzia Talamonti, Franco Fusi, Isacco Desideri, Pierluigi Bonomo, Lorenzo Livi, Stefania Pallotta

**Affiliations:** ^1^ Department of Clinical and Experimental Biomedical Sciences “Mario Serio” University of Florence Florence Italy; ^2^ Medical Physics Unit AOU Careggi Florence Italy; ^3^ Health Physics Unit AOU Careggi Florence Italy; ^4^ Radiation Therapy Unit AOU Careggi Florence Italy

**Keywords:** bending and shrinking, deformable image registration, deformable phantom, DIR algorithm accuracy assessment, similarity indices

## Abstract

A quantitative evaluation of the performances of the deformable image registration (DIR) algorithm implemented in MIM‐Maestro was performed using multiple similarity indices. Two phantoms, capable of mimicking different anatomical bending and tumor shrinking were built and computed tomography (CT) studies were acquired after applying different deformations. Three different contrast levels between internal structures were artificially created modifying the original CT values of one dataset. DIR algorithm was applied between datasets with increasing deformations and different contrast levels and manually refined with the Reg Refine tool. DIR algorithm ability in reproducing positions, volumes, and shapes of deformed structures was evaluated using similarity indices such as: landmark distances, Dice coefficients, Hausdorff distances, and maximum diameter differences between segmented structures. Similarity indices values worsen with increasing bending and volume difference between reference and target image sets. Registrations between images with low contrast (40 HU) obtain scores lower than those between images with high contrast (970 HU). The use of Reg Refine tool leads generally to an improvement of similarity parameters values, but the advantage is generally less evident for images with low contrast or when structures with large volume differences are involved. The dependence of DIR algorithm on image deformation extent and different contrast levels is well characterized through the combined use of multiple similarity indices.

## 1 INTRODUCTION

In the last few years deformable image registration (DIR) algorithms have become necessary tools in adaptive radiation therapy (ART) treatments. Radiotherapy plans may in fact need to be modified during the treatment course in order to be adapted to patient anatomical changes. Furthermore, there is an increasing number of retreatments that require effective instruments enabling reliable dose accumulation and contour propagation. The growing number of DIR implementations demands dependable methods to understand algorithms strengths and weaknesses. At the same time, as also suggested in the AAPM TG132^1^ recommendations, it is important to stress on all the possible implications behind DIR use in clinical routine. In the medical physics community there is a broad agreement on the necessity of an in‐deep assessment of DIR performances. Different approaches have been followed to perform such validation, involving the use of deformable phantoms, digital phantoms, and clinical patient data. Physical phantoms, mimicking realistic anatomy and containing internal deformable heterogeneities, have been manufactured and proposed for the validation of several algorithms in thorax, pelvis, and head and neck districts.^2^, ^3^, ^4^, ^5^, ^6^, ^7^ This approach is convenient but presents some limitations due to phantom unavailability for all the tasks to be tested and from the lack of a ground truth transformation. On the contrary, synthetic or digital phantoms, created by applying displacement vector fields to deform patient images, offer the possibility to perform a quantitative comparison between the ground truth and the algorithm‐created displacement vector fields.^8^, ^9^, ^10^, ^11^, ^12^, ^13^ This approach is interesting even if realistic deformations are quite difficult to be implemented in large regions. Finally, when patient data are used, DIR performances are usually assessed comparing anatomical marker positions^14^, ^15^, ^16^, ^17^, ^18^ in deformed and original images. Even if these tests offer useful clinical information, they are inadequate to fully describe algorithm behavior and to point out its limits.

Independently from the validation approach followed, several similarity indices can be used to assess registrations quality. The most commonly employed method consists of comparing the position of corresponding landmarks (points that can be easily recognized in all images) or the volume of corresponding structures in registered images. Each indicator provides helpful information for DIR accuracy quantification, but only the combined use of several indicators can fully characterize the registration quality pointing out errors in position or shape of registered structures.

A lot of different deformable algorithms have been developed in the last years and several studies have been proposed to highlight their strengths and weaknesses or to compare the performances of different algorithms. Several papers have been published on MIM DIR algorithm performances, mostly comparing MIM results with those obtained using other algorithms.^2^, ^7^, ^8^, ^11^, ^12^, ^13^, ^17^, ^18^ The impact of image characteristics (as contrast levels, noise, deformation etc.) on registration results has been studied but, as far as we know, no study examined different aspects separately.

In this work, we propose a multiparametric validation of the MIM‐Maestro DIR algorithm and Reg Refine tool (MIM Software, Cleveland, OH) considering some typical deformations that might appear in computed tomography (CT) studies during the course of head and neck radiotherapy treatments. For this purpose two real phantoms, simulating realistic deformations as neck bending and tumor shrinking were realized and CT studies were acquired after applying different deformations. Moreover, different contrast levels were artificially created modifying the CT values of one of the two phantom studies. Images of deformed phantoms were registered on original data with the DIR algorithm and the Reg Refine tool. Landmarks distances, Dice coefficients, Hausdorff distances, and maximum diameter differences between reference and registered images were used to quantify the algorithm capability in recovery reference positions and shape of points and structures.

## 2 MATERIALS AND METHODS

### 2.A Registration software

The DIR module of the MIM software uses a free‐form, intensity‐based algorithm to carry out CT to CT DIRs (http://downloads.mimsoftware.com.s3.amazonaws.com/brochures/MIM%20Maestro%20Unlimited%20Brochure.pdf, visited on November 2018). The deformable transformation is created starting from a rigid fusion of the initial image sets, through the minimization of a cost function that takes into account the image similarity and the physical likelihood of the transformation. During the optimization process, the image similarity has a higher weight compared to the physical likelihood, with the risk of producing unrealistic transformations.^7^ If the DIR results are not satisfactory, the user can refine the alignment using the Reg Refine tool.^11^, ^18^ In this case, boxes of adjustable dimensions are manually positioned in those regions where the alignment is not adequate and inside these boxes local rigid registrations are performed. A new DIR is then created combining the local registrations.

### 2.B Phantoms

Two phantoms were prepared to check DIR algorithm performances when bending and volume shrinking occur.

**Figure 1:**
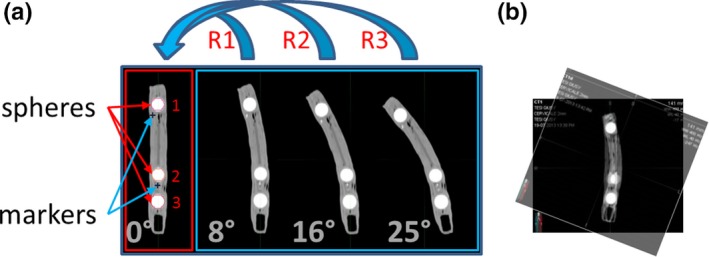
Coronal reconstructions of the phantom showing different bendings. The tree spheres and two of the seven internal markers are indicated in the 0° computed tomography study (a); initial rigid alignment for the R3 registration (b).

The Phantom 1, developed for *bending test,* is a stick made of modeling clay. Inside it seven small glass grains (diameter, d = 2 mm) and three glass spheres (d = 1.6 cm) acting as markers and reference structures were included. A deformation of the clay and deployment of the rigid structures is obtained by flexing the phantom (see Fig. 1). As the HU values of glass and modeling clay are different from those of real tissues, we modified them by using an in‐house written Matlab (MATLAB R2015b, The MathWorks Inc., Natick, MA, 2015) code to reproduce a realistic bone‐muscle contrast level between the spheres and the surrounding material (1000 and 30 HU, respectively). The background value was kept unchanged. The code rescales the HU by following a second‐degree polynomial curve that fits the HU of the background and the two new HU values.

**Figure 2:**
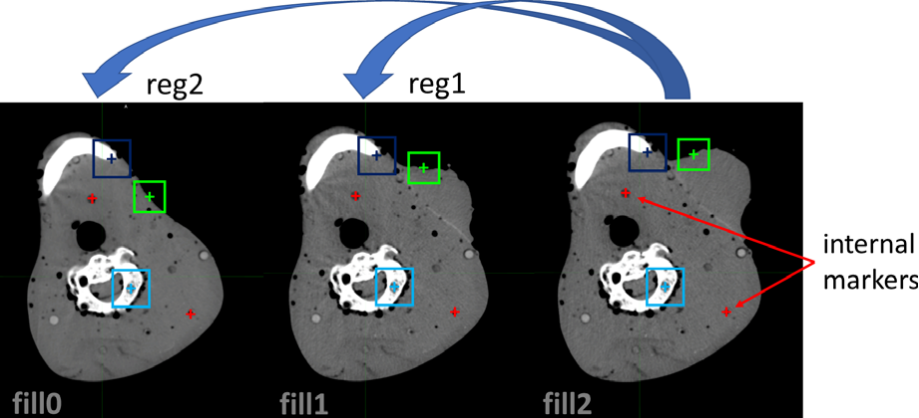
Images of the same phantom slice showing different tumor sizes; external contours and two internal markers are outlined. Colored boxes exemplify the boxes used by the Reg Refine tool that identify the regions forced to overlap during the refinement of the deformable registration.

The Phantom 2, developed for *shrinking test*, consists of a structure made by a rubber membrane filled with water and connected to a syringe with a small pipe, fixed between the head and the neck of an Alderson Rando phantom. Ultrasound gel was used to fill cavities to create a realistic mass protruding from the phantom. Volume variations were obtained filling the rubber membrane with different quantities of water. Eight phantom defects easily recognizable in all images were used as markers (see Fig. 2).

### 2.C Accuracy tests

A good DIR should be able to create registered images where points and structures have positions, shapes, and dimensions as similar as possible to the corresponding ones in reference images. In this work, these aspects were evaluated measuring the distances between corresponding marker positions and comparing shape and dimension of corresponding structures automatically segmented using the following similarity indices:

- the distance between centroids of corresponding markers (RM);
- the dice similarity coefficient (DSC) between corresponding contours (sensitive to translations and volume changes);
- the Hausdorff Distance (HD) between corresponding contours (sensitive to structures shape modifications).

When Phantom 1 was used, the distance between the centroid of corresponding spheres (R) and the absolute value of maximum diameter difference of corresponding spheres (DD) were also evaluated for each sphere.

Sensitivities of the methods used to evaluate the similarity indices were estimated by registering an image set with itself and evaluating RM, HD, DD, R, and DSC indices for each internal structure. For each index and phantom, the associated sensitivity was estimated as the maximum value obtained. For the DSC, the maximum difference from one was considered.

#### 2.C.1 Variable bending test

The DIR performances with respect to different degrees of bending were studied using Phantom 1 and acquiring CT studies with a Brilliance Big Bore CT scanner (Philips, Amsterdam, The Netherlands), (120 kV, 32 mAs, 2‐mm slice thickness and 0.24 × 0.24 mm^2^ pixel size). The CT study of the unmodified phantom [CT(0°)] was followed by three CT acquisitions where the phantom bending was increased progressively [see Fig. 1(a)]. The applied bending was a posteriori evaluated on CT images measuring the angle between the axes of the two extremities of the phantom that resulted 8°, 16°, and 25°, respectively. CT acquisitions acquired with increasing phantom bending [CT(8°), CT(16°), CT(25°)] were registered on CT(0°) obtaining R1, R2, and R3 registrations and corresponding registered datasets. In all cases the initial rigid registration was performed aligning spheres 1 and 3 as shown in Fig. 1(b). The three glass spheres were automatically segmented in CT(0°), R1, R2, and R3 using the same threshold (50% of max) and each marker centroid was localized in reference and deformed studies. HD, DSC, DD, R, and RM were finally measured.

#### 2.C.2 Variable contrast test

In order to assess whether DIR performances are influenced by different degrees of image contrast, the CT values of CT(0°), CT(8°), CT(16°), and CT(25°) were modified by using the in‐house written Matlab routine. Three contrast levels between the modeling clay and the spheres were created in each dataset while keeping unchanged the background value:

1. 30 and 1000 HU (30_1000) simulating muscle and bone;
−100 and 40 HU (−100_40) simulating fat and muscle;
3. 10 and 50 HU (10_50) simulating two different soft tissues.

For each created contrast level, three registrations were performed, and the same analysis previously described was performed on glass spheres and markers.

#### 2.C.3 Shrinking volume test

The CT images of Phantom 2 were acquired with the same scanner (120 kV, 2 mm slice thickness and 0.52 × 0.52 mm^2^ pixel size). Two CT studies CT(fill1) and CT(fill2) were performed after filling the membrane fixed to the Alderson Rando phantom with 25 and 50 ml of water. A CT study CT(fill0) of the same portion of the Alderson Rando phantom without adding the external structure was acquired (Fig. 2). CT(fill2) was registered on CT(fill1) and on CT(fill0) resulting in reg1 and reg2 registrations and corresponding registered studies. Volume differences (VD) between CT(fill2) and CT(fill1) and CT(fill2) and CT(fill0) were 25 and 50 ml, respectively. Each pair of studies was initially fused by optimizing the matching of bony structures. The phantom external contour and the mandible were segmented for 31 slices (6.2 cm) around the changing volume in references and registered studies and used to evaluate HD and DSC. RM between eight internal markers was also measured.

#### 2.C.4 Reg refine tests

All registrations were repeated using the Reg Refine tool. For Phantom 1, nine small boxes equally spaced along the phantom length and covering the entire phantom volume were used. For Phantom 2, the Reg Refine was applied using 25 boxes positioned near the changing volume to block fixed structures (such as bone structures) and to align the border of the protruding mass with the border of the reference image (see the exemplifying boxes in Fig. 2). In both cases, inside each box, an automatic rigid alignment was run before the box was locked. Positions and dimensions of the boxes were chosen following the suggestions found in reference^11^ that proposes to locate blocked boxes only in regions with evident deformation and to use larger boxes in case of low deformations and smaller boxes in highly deformed regions.

The same similarity indices were evaluated and compared to those previously obtained, in order to assess the Reg Refine contribution to the registration accuracy. Results were compared case by case, then outcomes from all tests were combined and a paired two‐sided Wilcoxon's signed‐rank tests with a significance level of 0.05 was performed for each set of similarity indices common to both phantoms to evaluate the statistical significance of differences between results obtained with and without the Reg Refine. All statistical analyses were performed with OriginPro (version 8, OriginLab Corporation, Northampton, MA).

## 3 RESULTS

### 3.A Sensitivity of the similarity assessment method

Sensitivities of the similarity indices RM, HD, DD, R, and DSC for Phantom 1 and Phantom 2 obtained registering two identical datasets are reported in Table 1. For DSCs, differences from 1 are reported. These values were compared with the differences between indices coming from different registrations to assess whether they were negligible or not case by case.

**Table 1:** Sensitivity of RM, Hausdorff distance (HD), DD, R, and dice similarity coefficient (DSC) similarity indices for Phantom 1 and Phantom 2

Phantom 1					Phantom 2		
RM (mm)	HD (mm)	DD (mm)	R (mm)	DSC	RM (mm)	HD (mm)	DSC
0.07	0.2	0.4	0.04	0.02	0.4	2	0.01

### 3.B Variable bending and variable contrast

**Figure 3:**
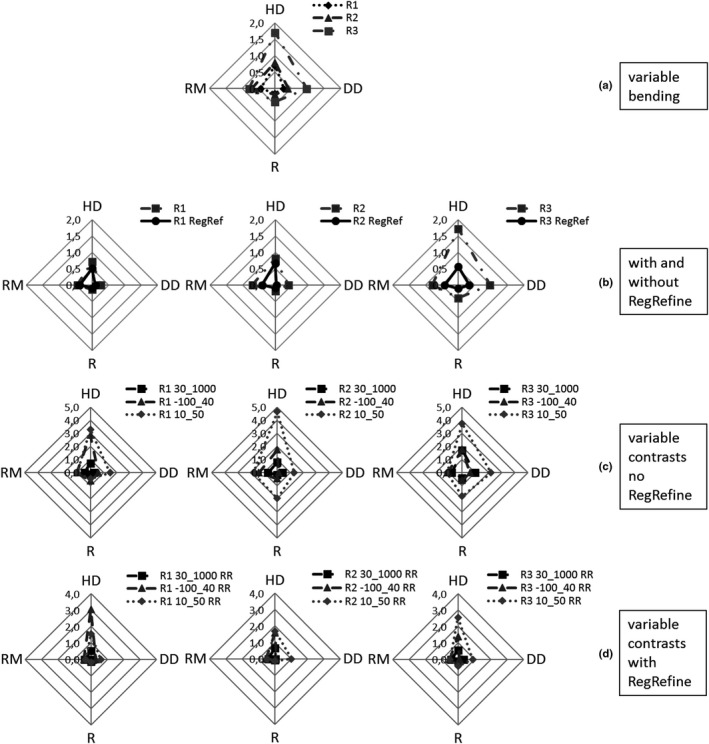
Spider graphs representing the mean values of Hausdorff distance, DD, R, and RM similarity indices. Results for R1, R2, and R3 are reported in (a) for variable bending test and in (c) for variable contrast test. Reg Refine contribution is described in (b) and (d) for variable bending and variable contrast test, respectively. Scales are in mm.

The DSC values for the three spheres of Phantom 1 calculated between CT(0°), R1, R2, and R3 are reported in Table 2. For the variable contrast test DSC values for the three spheres of Phantom 1 between CT(0°) and R3 are reported in the same table. DSC values demonstrate that registration between objects presenting increasing bending worsens. Moreover, when image contrast levels decrease, a worsening of DSC values is also observed as indicated by the results of 30_1000 and 10_50 tests. The mean values of HD, DD, and R (for the three spheres) and of RM (for the seven markers) for variable bending and variable contrast tests are reported in the spider graphs of Fig. 3. Smaller area indicates more accurate registration results. In Fig. 3(c) different contrast levels correspond to different polygon areas. In particular, larger areas can be noticed for R2 and R3 with contrast 10_50.

**Table 2:** Dice similarity coefficient (DSC) for the three spheres of Phantom 1 relative to the variable bending and variable contrast tests using (RR) or not (nRR) the Reg Refine tool. The values for which the difference between nRR and RR are greater than the sensitivity of the parameter are in bold

Phantom 1												
Contour	Variable bending — DSC						R3 — Variable contrast — DSC					
	R1		R2		R3		30_1000		−100_40		10_50	
	nRR	RR	nRR	RR	nRR	RR	nRR	RR	nRR	RR	nRR	RR
sphere1	0.97	0.98	0.96	0.98	**0.92**	**0.98**	**0.92**	**0.98**	0.95	0.96	**0.74**	**0.95**
sphere2	0.97	0.99	**0.96**	**0.99**	**0.88**	**0.98**	**0.88**	**0.98**	**0.83**	**0.96**	**0.83**	**0.96**
sphere3	0.99	0.99	0.98	0.99	0.98	0.98	0.98	0.98	0.97	0.95	0.97	0.97

### 3.C Shrinking volume

In Table 3, DSCs and HDs for mandible and external contours between reference and reg1 and reg2 are reported. DSCs for external contours are close to 1 in both cases and lower, but still close to 1, for mandible contours. HDs are close to measurement sensitivity (2 mm, see Table 1) for reg1 but become higher for reg2, especially for the external contour. Mean RM and standard deviations between corresponding markers in reference and deformed images for the eight internal markers of Phantom 2 are reported in Table 4. RMs are negligible for reg1. For reg2, RM values are higher and their distributions more scattered, but only for one marker RM exceeds 2 mm.

**Table 3:** Dice similarity coefficients (DSCs) and Hausdorff distance (HDs) for mandible and external contours for the shrinking volume test using (RR) or not (nRR) the Reg Refine tool. The values for which the difference between nRR and RR results is bigger than the sensitivity of the parameter are in bold. Differences in volume are 25 and 50 ml for reg1 and reg2, respectively

Phantom 2								
Contour	DSC reg1		DSC reg2		HD reg1 (mm)		HD reg2 (mm)	
	nRR	RR	nRR	RR	nRR	RR	nRR	RR
External contour	1.00	1.00	0.99	1.00	1	1	**9**	**3**
Mandible	0.98	0.98	**0.98**	**0.95**	2	2	**3**	**7**

**Table 4:** Mean and standard deviations of the distances between internal markers relative to the registrations of studies with increasing volume difference using (RR) or not (nRR) the Reg Refine tool. In bold are the values for which the difference between nRR and RR results is bigger than the sensitivity of the parameter

Phantom 2				
Markers distance	RM reg1 (mm)		RM reg2 (mm)	
	nRR	RR	nRR	RR
Mean	0.2	0.3	**0.8**	**2.8**
Standard deviation	0.3	0.3	0.8	3.4

### 3.D Reg refine test

In table 2, DSC values obtained applying the Reg Refine tool to all registrations performed on Phantom 1 are reported; comparing with values resulting from registrations not refined, in all cases, but one, DSCs are unchanged or increased. As noticeable from spider graphs in Fig. 3(b), the polygonal areas reduce when registrations are refined using the Reg Refine tool. Also, in the case of different contrast levels, the areas reduce if the Reg Refine tool is used [Fig. 3(d)] even though mean DD and HD are around 1 mm and higher than 1.5 mm, respectively. For all contrast levels, distances between correspondent points in reference, R2, and R3 exceed 2 mm for some markers; in all cases using the Reg Refine tool the number of these points is reduced or zeroed.

Concerning tests on Phantom 2, the use of Reg Refine gives worse results for mandible contours in reg2 as shown by DSC and HD values in Table 3. Using Reg Refine, mean RM remains unchanged for reg1, while it increases considerably for reg2 (Table 4), in this last case, for three markers, RM exceeds 2 mm abundantly.

Considering all tests together, the differences between similarity indices obtained with and without the use of Reg Refine resulted statistically significant: *P*‐values from Wilcoxon's tests for DSCs, HDs and RMs resulted respectively 0.01, 0.003 and 0.002.

## 4 DISCUSSION

In this work, several aspects of the MIM DIR process were separately investigated considering some typical deformations that might appear in CT studies during head and neck radiotherapy treatments. We investigated how the accuracy of the registrations depends on the structures’ shape and image contrast and whether the use of the Reg Refine tool improves the registration results. For these purposes, we developed two simplified physical phantoms to simulate neck bending and tumor volume shrinking, and we measured the quality of registration results using multiple indices. It is in fact only the combined use of multiple indices that permits to quantify all kind of errors in a registration process pointing out unrecovered translations and variations in shape or volume of structures depicted in reference and registered datasets.

From our tests, we have noticed that when objects are considerably deformed registration results get worse. The variable bending tests highlight that results get worse, as shown by increasing spider graph areas from R1 to R3, in case of a large difference in bending between reference and deformed images. Corresponding structures that in the initial rigid registration do not match [such as sphere 2 in our case, Fig. 1(b)] do not maintain their shape during the deformable registration process as demonstrated from DSCs values in Table 2. Moreover, HD and DD present a variability higher than that observed for corresponding markers (RM) and corresponding spheres (R) demonstrating that, in general, objects’ position is better reproduced than objects’ shape in registered images. It is worth noting that R is almost constant in all tests (variations lower than 0.5 mm) and lower than that of RM. In the first case, in fact, the intensity‐based registration algorithm is facilitated by the high contrast existing between spheres and clay. More generally, registration algorithm performances worsen when decreasing the image contrast, as demonstrated by DSC values in Table 2 and by large spider graphs area for low‐contrast images described in Fig. 3(c). Large bending and small contrast give the worst results. This is true not only for HD and DD but also for R, which results much higher than that obtained in high‐contrast images. The low contrast between spheres and clay lowers the registration algorithm capability to properly correct deformed objects.

By analyzing the results of the shrinking volume tests, it is possible to see that the registration quality also depends on the differences in phantom volume in the two image sets. In particular, the shape of the external contour is less accurately reproduced when larger volume differences are considered, as demonstrated by higher HD value for reg2 than reg1, visible in Table 3. On the contrary, DSC is quite insensitive to volume difference, probably due to the small relative volume changes of the external contour (maximum 50 over 700 ml).

**Figure 4:**
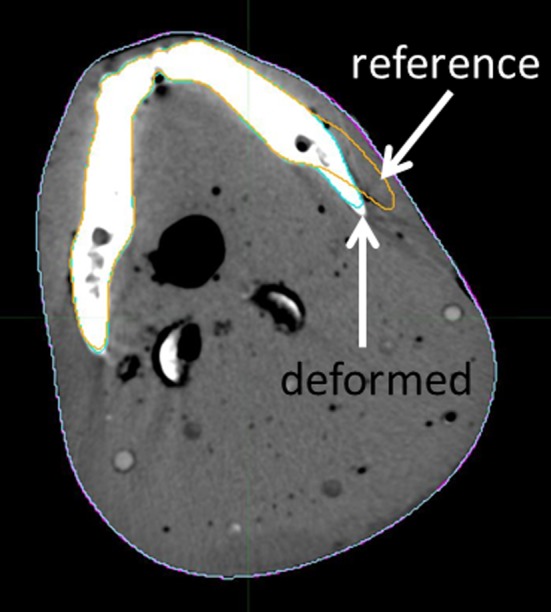
An axial slice of the reg2 deformed image set. External contour and mandible outlined in reference and deformed images are shown. While external contours are overlapped, an evident displacement of the two mandible contours is observable.

Finally, the Reg Refine tool leads generally to an improvement of registration quality. Analyzing case by case, we noticed that it happened in most cases, also in situations where the algorithm performances are originally poor. This is demonstrated by increased DSC values for sphere 2 when the Reg Refine tool was used (see Table 2). The advantage of Reg Refine is instead less relevant for images with low contrast especially in reproducing correctly structure's shape [see DD and HD of Fig. 3(d)] and for images with large volume differences (see Table 3). In this latter case, the use of blocked boxes that force the overlap of external contour, guarantees a better registration in this area but induces bony structure deformation (see Fig. 4). Moreover, the high value of mean markers’ distance (Table 4) indicates unrealistic deformations of homogeneous volumes where markers are embedded. In summary, the advantages of Reg Refine are relevant but only in localized areas around blocked boxes; far from these regions, benefits are lost.

Our results are in line with published data. It is widely accepted that in some cases DIR creates nonrealistic images^2^, ^7^ as we found for the transformation of the spheres in Phantom 1 for the 25° bending. Three studies concluded that the deformation quality is influenced by image noise^7^, ^8^, ^11^ leading to poorer performances when the registration involves images with low contrast.^2^, ^7^, ^11^ Howevern Pukala et al.^13^ found that registrations performed using the MIM algorithm in the head and neck area correspond to lower mean errors than four other commercial DIR algorithms. In the paper by Singhrao et al.,^2^ MIM DIR performances are investigated by using a head and neck deformable phantom and images from kV and MV tomographic imaging scanners, but the registration's accuracy is evaluated only using the distances between nonradiopaque markers in reference and deformed image sets. In one paper^11^, authors evaluated the contribution of the Reg Refine using lung images of real patients and digital head and neck phantoms with associated deformable vector fields. This thorough study demonstrated that the Reg Refine controlled by an expert user, lead to noticeable improvements both in the lung and in the head and neck areas. In another paper^8^, authors reported that the quality of the transformation depends on the image intensity but not on the deformation size, in contrast with our results. However, authors concluded that the results were better for localized deformations. Also in the works of Olteanu et al.^17^ and Broggi et al.^18^, it is demonstrated that DIR performance worsens when the volume difference of involved structure increases.

Published data and our work demonstrate that the performances of the algorithm depend on several factors and the quality of the registration can be different for different cases or anatomical areas. Particular attention should be paid to cases where the contrast among tissues is low and where large volume differences are present in localized regions.

With our study we have provided a method to measure MIM DIR performances in different challenging situations through the contemporary use of multiple parameters.

## 5 CONCLUSIONS

MIM DIR algorithm capability depends on the degree of deformation and contrast level of considered images. It does not give satisfactory results when structures show low‐contrast and high volume variations in localized regions. However, all analyzed registrations show an improvement at least in localized areas, when refined by using the Reg Refine tool. A method using a combination of parameters to evaluate the quality of the obtained registrations in different situations lead to a detailed analysis of the effectiveness of the MIM DIR algorithm.

## CONFLICT OF INTEREST

The authors certify that they have no affiliations with or involvement in any organization or entity with any financial interest or nonfinancial interest in the subject matter or materials discussed in this manuscript.
